# Pyridoxal-5′-Phosphate Promotes Immunomodulatory Function of Adipose-Derived Mesenchymal Stem Cells through Indoleamine 2,3-Dioxygenase-1 and TLR4/NF-*κ*B Pathway

**DOI:** 10.1155/2019/3121246

**Published:** 2019-11-25

**Authors:** Cong Li, Jinxian Huang, Huasu Zhu, Qing Shi, Dong Li, Xiuli Ju

**Affiliations:** ^1^Department of Pediatrics, Qilu Hospital of Shandong University, Shandong 250012, China; ^2^Stem Cell and Regenerative Medicine Research Center of Shandong University, Jinan, Shandong 250012, China

## Abstract

Adipose-derived mesenchymal stem cells (A-MSCs) are promising cellular therapies for the treatment of immune-mediated diseases. Non-gene editing technologies can improve the immune regulatory function of A-MSCs. Our preliminary experiments revealed that an active form of vitamin B6—pyridoxal-5′-phosphate (PLP)—plays an important role in regulating gene expression and cytokine secretion in A-MSCs *in vivo*. To further clarify the effect of PLP on receptors and cytokines related to the immune regulatory function of A-MSCs, a series of experiments were designed to verify the relationships between PLP and A-MSCs *in vitro*. Initially, A-MSCs were obtained, and cytokine secretion and the expression of IDO1, NF-*κ*B, and Toll-like receptors in PLP-stimulated A-MSCs were evaluated. In addition, coculture was used to detect A-MSCs-mediated apoptosis of CD3^+^CD8^+^ T lymphocytes. These results showed that A-MSCs stimulated with PLP were highly proliferative, consistent with their pluripotent capacity. Further, the surface receptors TLR3, TLR4, IDO1, and NF-*κ*B were upregulated, while TLR6 was downregulated. Concurrently, A-MSCs preconditioned with PLP had the greatest inhibitory effect on CD3^+^CD8^+^ T lymphocyte proliferation, indicating that PLP altered the immune regulatory function of A-MSCs through the regulation of TLRs and IDO1 expression.

## 1. Introduction

Based on previous studies, adipose-derived mesenchymal stem cells (A-MSCs) are known to have immunomodulatory functions [1] and have been reported in clinical applications, such as the treatment of systemic lupus erythematosus (SLE) [2], graft versus host disease (GVHD) [3], and systemic sclerosis [4]. The immunomodulatory function of A-MSCs is positively correlated with its dosage; however, high-dose mesenchymal stem cells cause an increase in adverse reactions such as increased acute pulmonary embolism [5]. Therefore, it is essential to improve the immune regulation of individual A-MSCs. Studies have shown that Toll-like receptor 3 (TLR3) [6], indoleamine 2,3-dioxygenase-1 (IDO1) [7], and prostaglandin E2 (PGE2) [8] are closely related to A-MSC immunomodulation, which can promote tryptophan metabolism and increase the proportion of Treg cells [9]. Upregulation of TLR3 and IDO1 can enhance the immunomodulatory function of A-MSCs [10]. Vitamin B6, including pyridoxal, pyridoxamine, and pyridoxine, exists as a phosphate in the body, which is associated with immunomodulatory function [11]. While all three species can be phosphorylated, pyridoxal-5′-phosphate (PLP) is the biologically most active form and is used as a cofactor for many important enzymatic reactions. In previous studies, we found that PLP inhibited the proliferation of T lymphocytes and reduced the inflammatory response [12]. Further, we found that certain concentrations of PLP could enhance A-MSCs proliferation, TLR3 and TLR4 expression, and IDO1 secretion. Simultaneously, the immunomodulatory function of A-MSCs to reduce the local inflammatory response was changed. Therefore, in this study, we further explored the role of PLP in the immunomodulatory function of A-MSCs.

## 2. Materials and Methods

### 2.1. A-MSCs Culture

Adipose tissue derived from abdominal liposuction surgery was provided by the Department of Plastic and Cosmetic Surgery, Qilu Hospital of Shandong University, Jinan, Shandong, China. Informed written consent was obtained for tissue use. Further, the use of adipose tissue was approved by the Ethics Committee of Shandong University Qilu Hospital (Jinan, China). The adipose tissue was cut into small pieces (1 mm^3^) and digested with 0.1% type I collagenase (eBioscience, San Diego, CA, USA). The digested products were resuspended and plated in a plastic flask at 1000 cells/cm^2^ using *α*-MEM medium (TBD, TBD32561, China) supplemented with 10% foetal bovine serum (FBS, Gibco, 30044333, USA) and 1% penicillin-streptomycin (Gibco, USA) and incubated at 37°C in a moist dark environment containing 5% CO_2_.

### 2.2. Phenotypic Analysis

Flow cytometry was performed to characterise A-MSCs. The following cell surface epitopes were detected using anti-human CD29 (eBioscience, 4303570, 1 : 20), CD31 (eBioscience, 4303600, 1 : 20), CD44 (eBioscience, 4330029, 1 : 20), CD45 (eBioscience, 4343394, 1 : 20), CD73 (eBioscience, 4344363, 1 : 20), CD90 (eBioscience, 4307303, 1 : 20), and CD105 (eBioscience, 1983608, 1 : 20). Briefly, 1 × 10^6^ cells were stained with 0.125 *μ*g of each specific fluorescence-labelled antibody in 100 *μ*L of phosphate-buffered saline (PBS) for 20 min at 25°C. Flow cytometry was performed with the Guava easyCyte 6HT (EMD Millipore, Billerica, MA, USA), and the data were examined using the Guava InCyte software (3.1 version, EMD Millipore).

### 2.3. Osteogenic, Adipogenic, and Chondrogenic Differentiation

To investigate the differentiation potential of fibroblast-like cells, P3 cells were cultured under conditions appropriate for inducing differentiation of each lineage. Cells were seeded at a density of 2 × 10^4^ cells/cm^2^. All groups were cultured for 24 days, and the differentiation medium was changed every 3 days (Table 1). At the same time, control cells were cultured in normal medium. At the end of 24 days, all cells were collected for subsequent analysis.

### 2.4. Cell Treatment and Microfluidic Chip Fabrication

The microfluidic chip was used in cell treatment, and the microchannel network was composed of an upstream gradient-forming unit and a downstream cell culture unit. The culture cell of the cell culture unit was connected to the central outlet via a channel, according to previously described methods [13]. Cell culture medium was prepared with different PLP (MACKLIN, 54-47-7, China) concentrations (ranging from 0 to 1000 ng/mL). Cell proliferation was detected by CCK-8 (BestBio, BB-4202-1, China), and the number of living cells was calculated at different PLP concentrations. The cells were aspirated into a microinjection pump prior to injection, and the capillary was connected to transfer to the cell tank at the cell flow rate of 0.2 *μ*L/s within 20 min. Following this, the water outlet was closed, and the chip was placed in the incubator for 7 h at 37°C, 5% CO_2_. Further, the water outlet was opened, and 0 and 1000 ng/mL of PLP were administered at the two inlets to perfuse the culture solution at a rate of 20 *μ*L/h when cell adherence was completed. After 48 h, 10 mL of CCK-8 was slowly perfused from the inlet at a flow rate of 50 *μ*L/h until the medium was completely discharged, and the inlet and outlet were closed. The perfused chip was incubated at 37°C, 5% CO_2_ for 4 h, and then directly inserted into the cell tank with a 1 mL syringe. Cell culture supernatant was taken and placed in a 96-well plate to measure absorbance at 450 nm.

### 2.5. Apoptosis Detection

A-MSCs were washed twice with cold PBS (phosphate-buffered saline) after exposing to different concentrations of PLP for 48 h and then resuspended in 1 × Binding Buffer at a concentration of 1 × 10^6^ cells/mL. All operations were carried out according to the FITC Annexin V Apoptosis Detection Kit (BD, 556547, USA) instructions and analysed by flow cytometry within 1 h.

### 2.6. Coculture of A-MSCs with T Lymphocytes

Human umbilical cord blood samples (*n* = 3, woman) were obtained from women undergoing full-term deliveries between January and May 2018 at the Department of Obstetrics at Qilu Hospital of Shandong University (Jinan, China), and informed written consent was obtained from all patients. The use of umbilical cord blood was approved by the Ethics Committee of Shandong University Qilu Hospital (Jinan, China). Human umbilical cord blood mononuclear cells (hUCB-MNCs) were isolated and collected using lymphocyte separation medium (TBD, LTS1077, China), and hUCB-MNCs were labelled with CFSE (BD Horizon™, 565082, USA) cultured in RPMI 1640 medium (Gibco, 11875, USA) containing 10% FBS, anti-CD3 mAb (eBioscience™, 16-0037-85, USA) to a final concentration of 100 ng/mL, and PHA-P (Sigma-Aldrich, L8754, USA) to a final concentration of 10 *μ*g/mL for 48 h. A-MSCs were stimulated with 1–1000 ng/mL PLP for 48 h. Following this, hUCB-MNCs were cocultured with stimulated A-MSCs at a ratio of 5 : 1 (hUCB-MNC : A-MSC), and the non-cocultured group with A-MSCs was set as the blank group. hUCB-MNC was marked with anti-human CD3 PE-Cyanine7 (eBioscience, 25-0037-41, USA) and anti-human CD8 PE (eBioscience, 25-0037-41, USA). The fluorescence intensity of CFSE was measured by flow cytometry to compare the cell proliferation of different groups after 3 days of coculture. To consider whether PLP residues affected T lymphocytes, lymphocytes were cultured in different PLP concentrations (0 ng/mL, 2 ng/mL, 4 ng/mL, 6 ng/mL, 8 ng/mL, and 10 ng/mL PLP) in RPMI 1640 medium supplemented with 10% FBS. Following this, the proliferation, apoptosis, and proportion of CD3^+^CD8^+^ T lymphocytes were detected after 48 h.

### 2.7. PCR Analysis of TLRs, NF-*κ*B, and IDO1 Expression

The upper PDMS membrane was removed gently with tweezers, and the cells were digested to extract RNA for reverse transcription sequencing after continuous perfusion culture for 48 h. Total RNA was extracted from 0 to 1000 ng/mL PLP-stimulated A-MSCs using TRIzol reagent (Invitrogen, 15596018, Carlsbad, CA, USA) and subsequently reverse transcribed into cDNA. qRT-PCR analysis was performed to measure mRNA expression of IDO1, TLR3, Toll-like receptor 4 (TLR4), Toll-like receptor 6 (TLR6), and housekeeping gene glyceraldehyde-3-phosphate dehydrogenase (GAPDH). qRT-PCR was performed using the Real-Time Thermal Cycler (Analytik Jena AG, qTOWER3G, Germany), and 0 ng/mL PLP-treated cells served as the control group. Reverse transcription was performed using PCR Kits from TOYOBO (Code Nos. FSQ-101 and QPK-201), according to the manufacturer's protocol. The primer sequences used for quantitative RT-PCR are listed in Table 2.

### 2.8. Western Blot Analysis of TLRs and IDO1 Expression

The cells were placed in cold RIPA solution (Solarbio, C0065) with PMSF (Solarbio, P0100) after washing the cells twice with phosphate buffer. Subsequently, they were pyrolysed with a protease inhibitor mixture for 30 min. The supernatant was centrifuged and harvested to analyse the protein concentration using the BCA Protein Assay Kit (BestBio, A045-4). Immunoblotting was conducted using the SDS-PAGE electrophoresis system. Briefly, 25 *μ*L of sample was loaded and electrophoresed on a 10% SDS reducing gel. Blots were blotted onto a polyvinylidene fluoride membrane and incubated with primary antibodies against TLR3 (1 : 1000, Abcam, ab13915, USA), TLR4 (1 : 500, Abcam, ab13556), TLR6 (1 : 1000, Proteintech, 22240-1-AP), IDO1 (1 : 500, Abcam, ab55305), and NF-*κ*B (1 : 500, Abcam, ab14059) overnight at 4°C. On the following day, the blot was washed three times with TBST and incubated with horseradish peroxidase-conjugated (HRP-conjugated) AffiniPure Goat anti-Mouse IgG (H+L) (1 : 5000, Proteintech, SA00001-1, USA) or HRP-conjugated AffiniPure Goat anti-Rabbit IgG (H+L) (1 : 5000, Proteintech, SA00001-2). Further, the blot was kept at 25°C for 1 h in TBST. Finally, proteins on the washed membrane were visualised using the TBST (Solarbio, T1082).

### 2.9. IDO1 and PGE2 Detection by ELISA

A-MSCs inoculated at 1 × 10^6^ cells/cm^2^ were exposed to different concentrations of PLP for 48 h, and the levels of culture supernatant of PGE2 (Cayman, 514010, USA) and IDO1 (RayBiotech, ELH-IDO, USA) at 48 h after treatment were detected by ELISA, according to the manufacturer's instructions.

### 2.10. TLR3/4 Inhibition and Supernatant Kynurenic Acid Detection

The TLR3 inhibitor CU CPT 4a (ApexBio, B5752, USA) and TLR4 inhibitor TAK-242 (ApexBio, A3850, USA) were separately added to the *α*-MEM cell medium containing 10% FBS to a final concentration of 10 *μ*M. The supernatant was removed and repeatedly washed with 0.9% NaCl after a 24 h incubation with A-MSCs. Following this, 0–1000 ng/mL PLP were added to stimulate A-MSCs for 48 h. Simultaneously, cells without inhibitor stimulation were used as control groups. Lymphocytes were cocultured with PLP-stimulated A-MSCs for 48 h, and the supernatant and cells were collected to measure the ratio of CD3^+^CD8^+^ T lymphocytes. The level of kynurenic acid in the supernatant from blank groups was determined using a human kynurenic acid kit (JSBOSSEN, BS-E7827H2, China).

### 2.11. Statistical Analyses

All data are presented as mean ± SEM of three separate experiments. Statistical analyses were assessed by one-way analysis of variance (ANOVA), which was used to compare multiple groups. For significant ANOVA results, the Bonferroni test was performed. Statistical analyses were performed using SPSS Statistics 21.0 (SPSS Inc., Chicago, IL, USA). Differences were considered statistically significant at a probability level less than 0.05 (*P* < 0.05).

## 3. Results

### 3.1. Characterisation of A-MSCs

**Figure 1:**
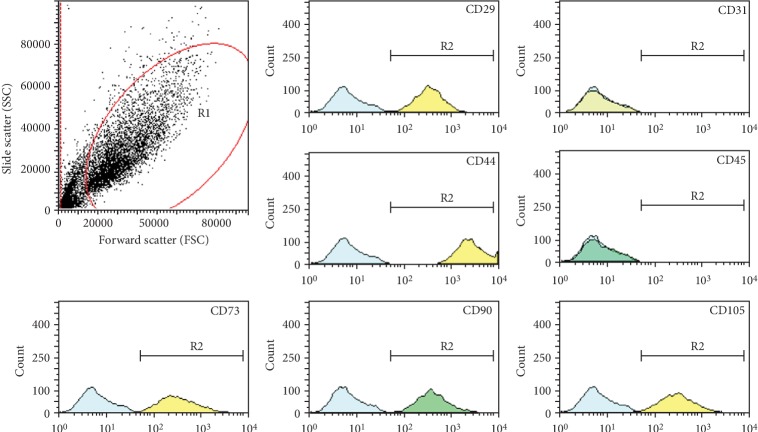
Phenotypic identification of A-MSCs. The isolated cultured cells were stained with MSC surface markers CD29, CD31, CD44, CD45, CD73, CD90, and CD105.

Adherent A-MSCs were obtained by enzymatic digestion, and they could rapidly proliferate *in vitro*. These isolated cells strongly expressed MSC surface markers, CD29, CD44, CD73, CD90, and CD105, and did not express the blood cell markers CD31 and CD45 (Figure 1).

These cultured cells exhibited a typical spindle-shaped, fibroblast-like morphology (Supplementary [Supplementary-material supplementary-material-1]C) and could differentiate into osteoblasts, adipocytes, and chondrocytes under the corresponding induction conditions. The cells were stained with Oil Red O (Supplementary [Supplementary-material supplementary-material-1]A), Alizarin red (Supplementary [Supplementary-material supplementary-material-1]B), Alcian blue (Supplementary [Supplementary-material supplementary-material-1]D), and Safranin O Green Cartilage Stain (Supplementary [Supplementary-material supplementary-material-1]E). Control group cells remained the shape of MSCs with negative staining marks (Supplementary [Supplementary-material supplementary-material-1]F).

### 3.2. PLP Reduced Apoptosis of A-MSCs

**Figure 2:**
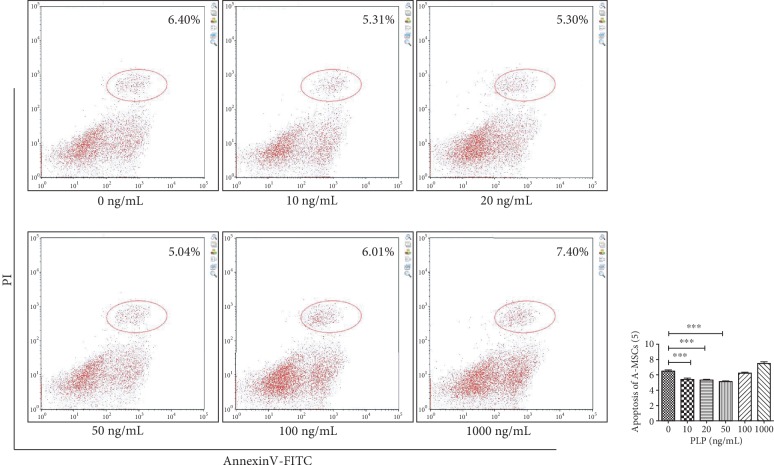
Apoptosis of A-MSCs as detected by flow cytometry. A-MSCs were stimulated with 0 ng/mL, 10 ng/mL, 20 ng/mL, 50 ng/mL, 100 ng/mL, and 1000 ng/mL PLP for 48 h, and the apoptosis rate of A-MSCs decreased significantly at 50 ng/mL PLP.

Apoptosis was detected by flow cytometry in A-MSCs exposed to 0–1000 ng/mL PLP. The results showed that PLP reduced apoptosis of A-MSCs in a concentration-dependent manner range, especially at the concentration of 50 ng/mL (Figure 2).

### 3.3. CD3^+^CD8^+^ T Lymphocyte Proliferation

**Figure 3:**
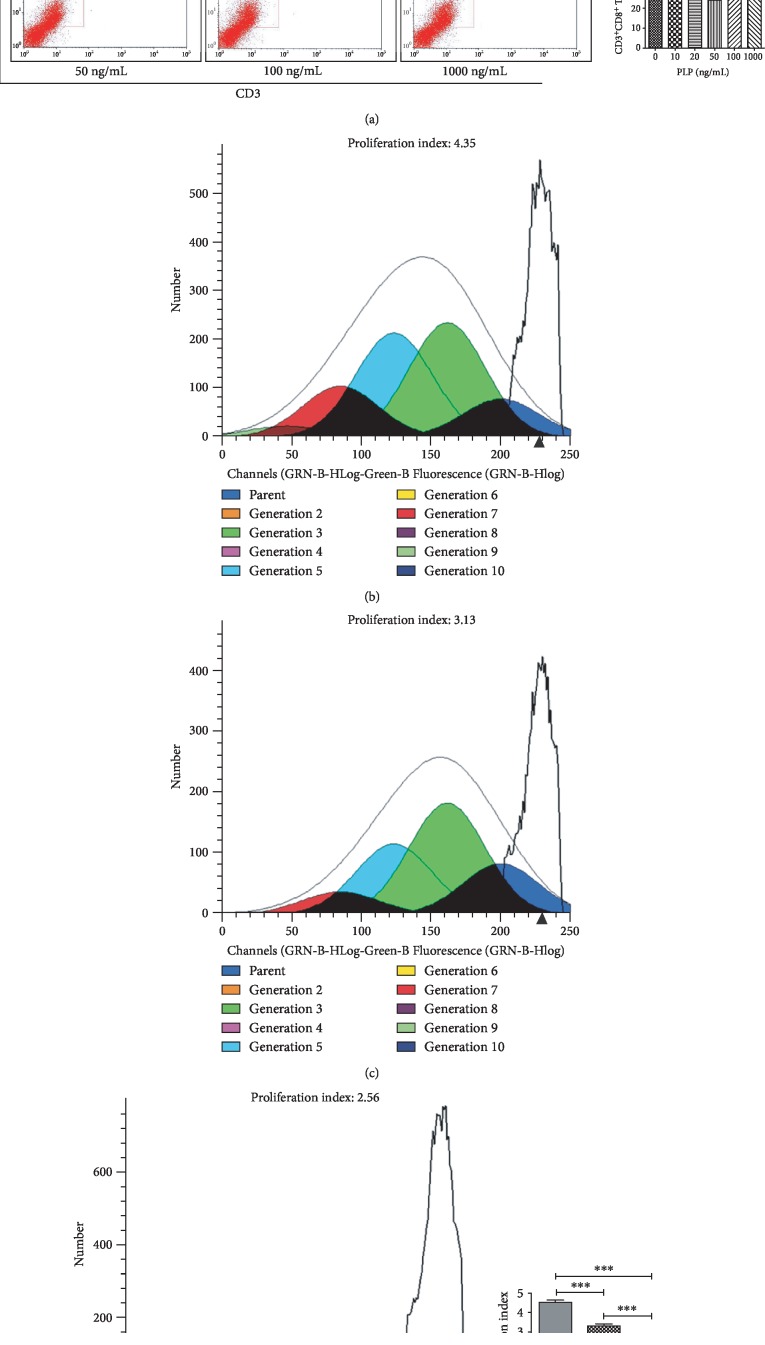
Flow cytometric analysis of the proportion and proliferation ability of CD3^+^CD8^+^ T lymphocytes. (a) Proportion of CD3^+^CD8^+^ T lymphocytes. (b) Blank group. (c) Control group, lymphocytes cocultured with A-MSCs stimulated at 0 ng/mL PLP. (d) Treated group, lymphocytes cocultured with A-MSCs stimulated at 50 ng/mL PLP.

Using the Transwell coculture technique, A-MSCs preconditioned with 50 ng/mL PLP showed the greatest inhibitory effect on CD3^+^CD8^+^ T lymphocyte proliferation. Further, T lymphocyte proliferation by A-MSCs pretreated with 50 ng/mL PLP was assessed, which demonstrated that PLP-treated A-MSCs reduced the proportion of lymphocytes by increasing their ability to inhibit lymphocyte proliferation (Figure 3). The proliferation index and ratio of CD3^+^CD8^+^ T lymphocytes were significantly higher than those cocultured with MSCs when lymphocytes were cultured at low concentrations in PLP alone, and there was no difference between concentrations (Supplementary [Supplementary-material supplementary-material-1], [Supplementary-material supplementary-material-1]). Similarly, there was no significant difference in apoptosis, indicating that low concentrations of residual PLP did not affect apoptosis and proliferation of CD3^+^CD8^+^ T lymphocytes (Supplementary [Supplementary-material supplementary-material-1]).

### 3.4. Cell Proliferation and Gene Expression in Microfluidic Chip-Cultured Cells

**Figure 4:**
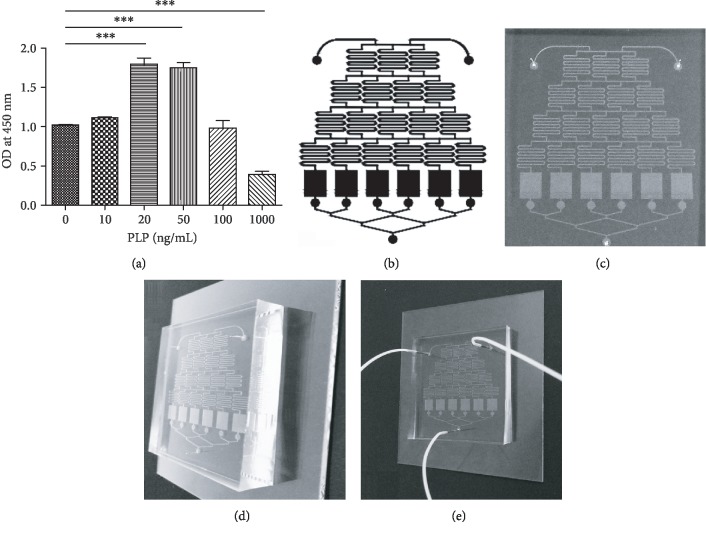
Proliferative capacity of A-MSCs after 48 h under different PLP stimulations using the CCK-8 kit. (a) The most proliferative ability appeared in 20–50 ng/mL; however, it was inhibited at 1000 ng/mL. (b) Design diagram. (c–e) Perfusion images of microfluidic chip.

Using the CCK-8 method, 1–100 ng/mL demonstrated the strongest proliferative capacity after 48 h of perfusion (Figure 4(a)). Further, the number of viable cells was the highest at 50 ng/mL, and the number was significantly reduced at 1000 ng/mL, indicating that this concentration was cytotoxic (Supplementary [Supplementary-material supplementary-material-1]). Further, RT-PCR was performed on cells cultured in microfluidic chips within this concentration range to detect the expression of TLR3, TLR4, TLR6, IDO1, and NF-*κ*B (Figures 4(b)–4(e)).

**Figure 5:**
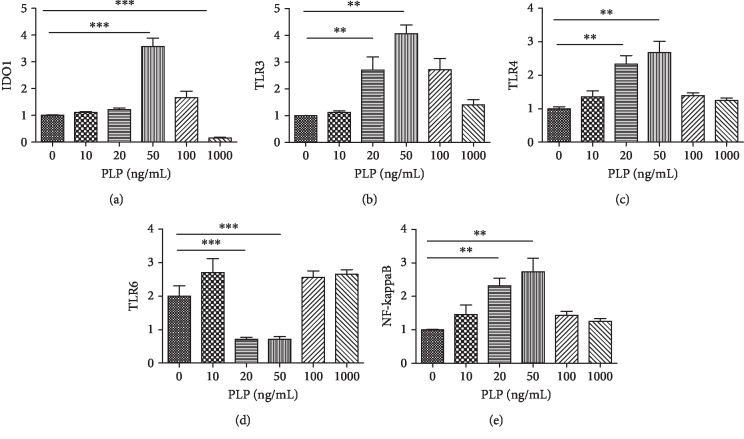
Relative gene expression of IDO1 (a), TLRs (b–d), and NF-*κ*B (e) stimulated with 0 ng/mL, 10 ng/mL, 20 ng/mL, 50 ng/mL, 100 ng/mL, and 1000 ng/mL PLP after 48 h.

After 48 h of stimulation with different concentrations of PLP, RT-PCR showed altered expression of the surface receptors TLRs and IDO1 in A-MSCs. Both TLR3 and TLR4 were upregulated; however, TLR6 was downregulated at 20-50 ng/mL PLP stimulation, compared to the control group. There was no significant difference at other concentrations. For the IDO1 receptor, it was downregulated at 1000 ng/mL and upregulated at 50 ng/mL PLP (Figure 5).

### 3.5. TLRs, IDO1, and NF-*κ*B Protein Expression in A-MSCs

**Figure 6:**
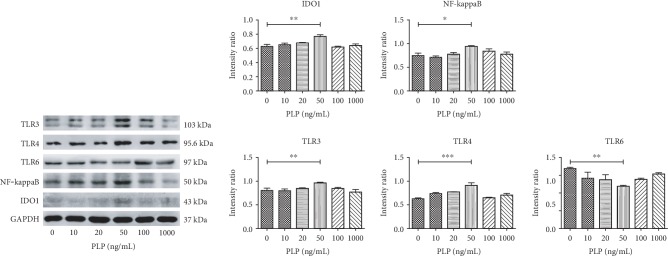
Western blot detection of immune-related proteins TLRs, IDO, and NF-*κ*B stimulated by different PLP concentrations. TLR3, TLR4, NF-*κ*B, and IDO had the highest protein expression levels, while TLR6 protein expression was the lowest at 50 ng/mL PLP.

Western blot analysis showed that the expression of TLRs, IDO1, and NF-*κ*B in A-MSCs was consistent with the PCR results. Specifically, the surface receptors TLR3, TLR4, IDO1, and NF-*κ*B were upregulated, while TLR6 was downregulated with PLP (50 ng/mL**)** stimulation compared to the control group, and other concentrations showed no significant difference (Figure 6).

**Figure 7:**
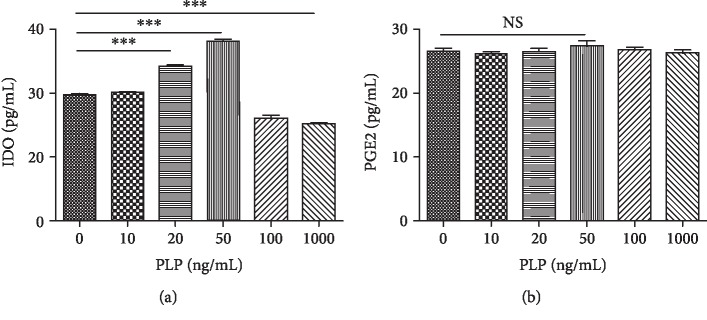
Secreted IDO1 and PEG2 levels in the supernatant, as measured by ELISA. (a) IDO1 level increased at 20–50 ng/mL yet decreased at 1000 ng/mL. (b) PGE2 level was not significantly different at each concentration.

Further, cytokine secretion in A-MSCs was examined after 48 h of stimulation with different concentrations of PLP. IDO1 levels were significantly increased at 50 ng/mL, while the PGE2 level did not differ significantly under stimulation at each concentration (Figure 7).

### 3.6. Immunofluorescence Expression of IDO1 and TLR3

**Figure 8:**
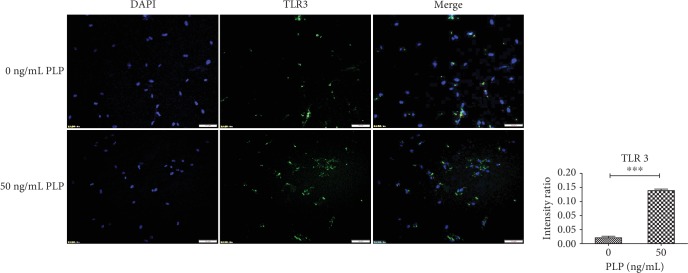
A-MSC stimulation with 0 ng/mL and 50 ng/mL PLP for 48 h. Further, TLR3 expression was detected by immunofluorescence. Scale bar = 100 *μ*m.

**Figure 9:**
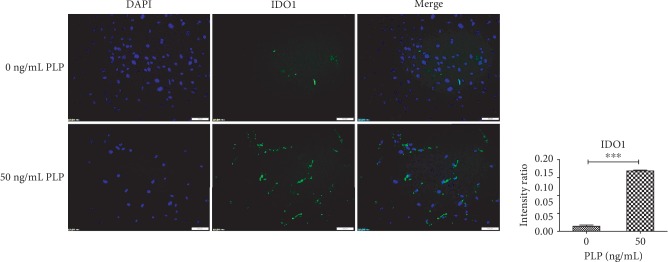
A-MSC stimulation with 0 ng/mL and 50 ng/mL PLP for 48 h. Further, IDO1 expression was detected by immunofluorescence. Scale bar = 100 *μ*m.

Surface protein receptor TLR3 and secreted IDO1 expression in A-MSCs were examined by immunohistochemistry. Compared with 0 ng/mL PLP, TLR3 and IDO1 expression levels in A-MSCs stimulated by 50 ng/mL PLP were higher (Figures 8 and 9).

### 3.7. Inhibition of CD3^+^CD8^+^ T Lymphocytes after TLR3/4 Inhibition

**Figure 10:**
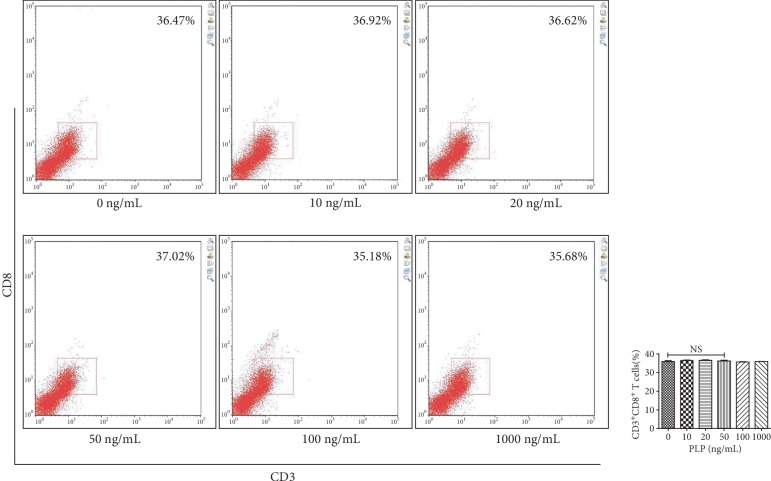
Flow cytometric analysis of the proportion of CD3^+^CD8^+^ T lymphocytes after A-MSCs coculture with TLR3 inhibitor. There was no significant difference in the proportion of CD3^+^CD8^+^ T lymphocytes between concentrations.

**Figure 11:**
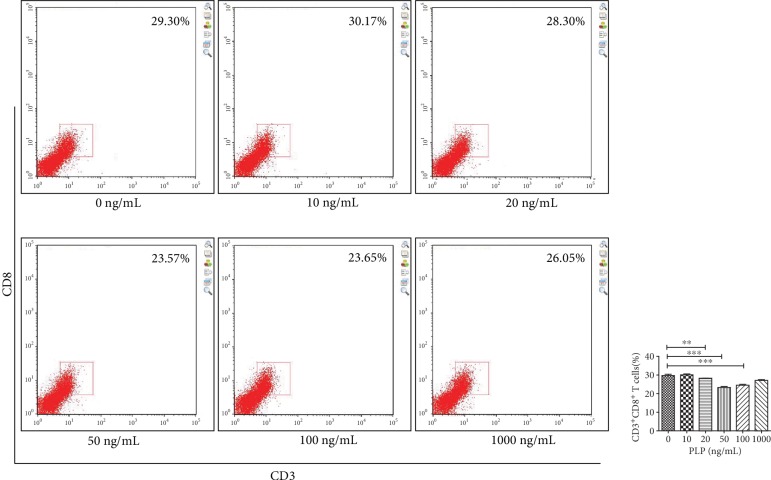
Flow cytometric analysis of the proportion of CD3^+^CD8^+^ T lymphocytes after A-MSCs coculture without inhibition. The proportion of CD3^+^CD8^+^ T lymphocytes decreased significantly at 20 ng/mL, 50 ng/mL, and 100 ng/mL compared to 0 ng/mL.

**Figure 12:**
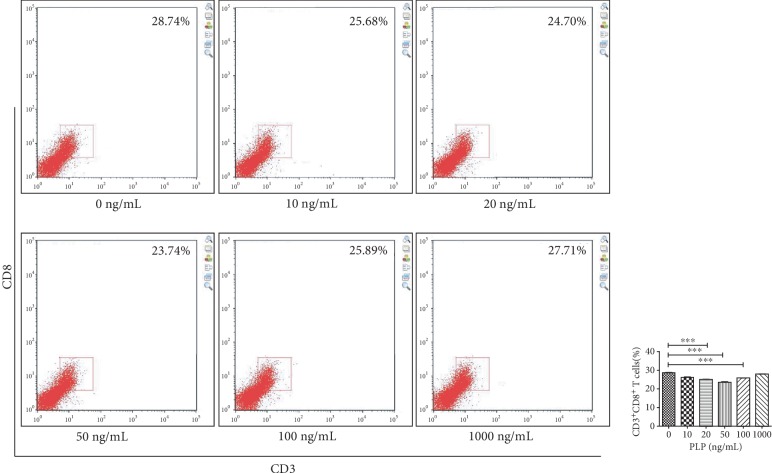
Flow cytometric analysis of the proportion of CD3^+^CD8^+^ T lymphocytes after A-MSCs coculture with TLR4 inhibitor. The proportion of CD3^+^CD8^+^ T lymphocytes decreased significantly at 50 ng/mL compared to 0 ng/mL.

**Figure 13:**
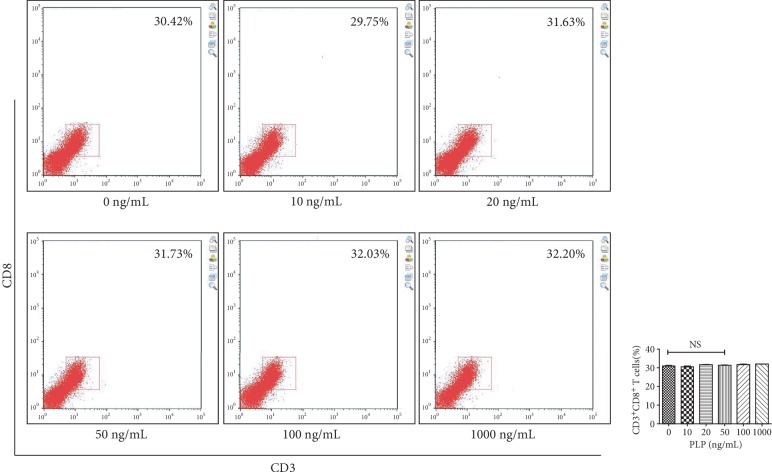
Flow cytometric analysis of the proportion of CD3^+^CD8^+^ T lymphocytes after A-MSCs coculture with TLR3 and TLR4 inhibition. There was no significant difference in the proportion of CD3^+^CD8^+^ T lymphocytes between concentrations.

**Figure 14:**
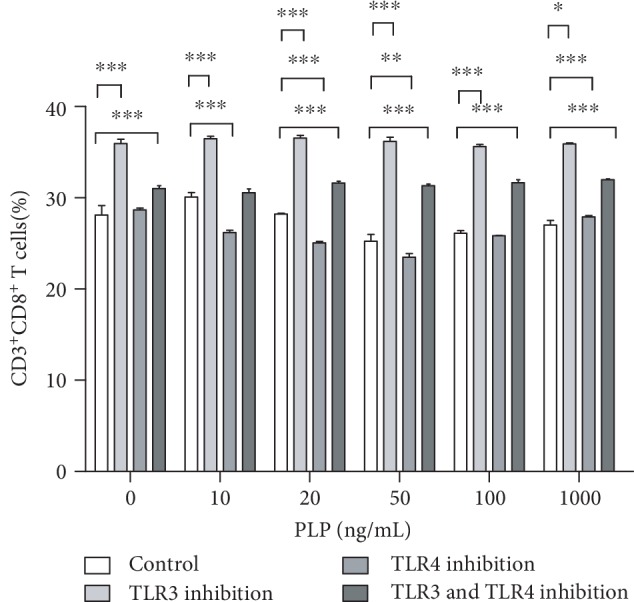
Proportion of CD3^+^CD8^+^ T lymphocytes after coculture of A-MSCs with or without inhibitor. The proportion of CD3^+^CD8^+^ T lymphocytes was significantly lower in the TLR4 inhibitor group at each concentration than the control group. While the concentrations in the TLR3 and TLR4 coinhibition groups were significantly lower than those in the control group, there was no significant difference from the TLR4 group.

TLR3 and TLR4 inhibitors were used alone or together to detect the effect of PLP on TLR3 and TLR4. We found that the CD3^+^CD8^+^ T lymphocytes were mostly in the 50 ng/mL group, compared to the control group, when the TLR3 inhibitor was used alone, which was not obvious at other concentrations (Figures 10 and 11). The proportion of CD3^+^CD8^+^ T lymphocytes in each concentration group was inhibited in the TLR4 alone inhibition group and was most significant in the 50 ng/mL group (Figure 12). Further, the proportion of CD3^+^CD8^+^ T lymphocytes in each concentration group decreased compared to TLR4 alone inhibition group, when TLR3 and TLR4 were coinhibited (Figures 13 and 14).

### 3.8. Increased Kynurenic Acid after Coculture

**Figure 15:**
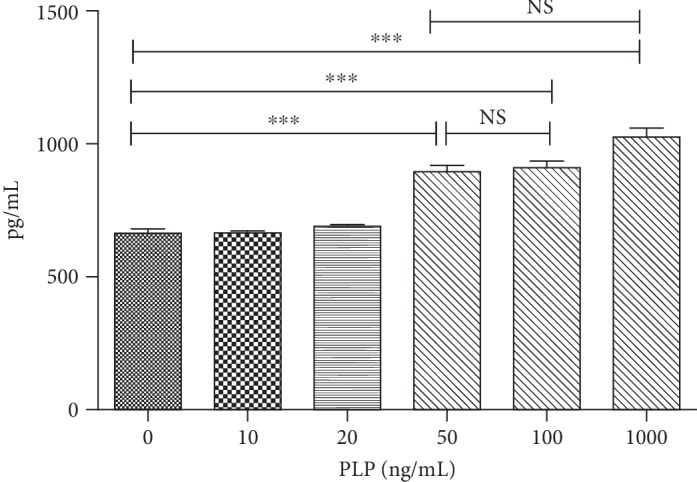
Kynurenic acid levels in supernatant of T lymphocytes coculture with A-MSCs stimulated by 0 ng/mL, 10 ng/mL, 20 ng/mL, 50 ng/mL, 100 ng/mL, and 1000 ng/mL PLP. The concentration of kynurenic acid was maximum at 50 ng/mL and 100 ng/mL. There was no significant difference in kynurenic acid levels at 1000 ng/mL and 50 ng/mL.

The levels of kynurenic acid in supernatants cocultured with A-MSCs stimulated with different concentrations of PLP were detected by ELISA. The kynurenic acid level of the coculture supernatant at the concentration of 50 ng/mL PLP was almost the same as that of 100 ng/mL and 1000 ng/mL, which was significantly higher than the non-PLP-stimulated group. Therefore, considering the cytotoxicity, 50 ng/mL was chosen as the most suitable concentration to upregulate immunomodulatory function (Figure 15).

## 4. Discussion

A-MSCs are adipose-derived pluripotent stem cells. Discovery of these cells allows a large number of cells with strong proliferative capacity to be extracted from adipose tissue [14, 15]. Further, A-MSCs can maintain pleiotropic differentiation ability and immunomodulatory function. In this study, A-MSCs were cultured by enzymatic digestion. These cells could proliferate rapidly *in vitro*. They also highly expressed MSC surface markers CD29, CD44, CD73, CD90, and CD105 yet showed no expression of blood cell-related markers CD31 and CD45. Further, these cells could differentiate into osteoblasts, adipocytes, and chondrocytes under corresponding induction conditions. To improve the immunosuppressive ability of A-MSCs, a microfluidic chip preexperiment was used to investigate whether 50 ng/mL PLP could stimulate proliferation of A-MSCs. Further coculture experiments showed that pretreatment of A-MSCs with 50 ng/mL PLP could significantly inhibit CD3^+^CD8^+^ T lymphocyte proliferation without apoptosis. This concentration of PLP could upregulate TLR3, TLR4, and IDO1 expression without any concomitant upregulation of PGE2.

PLP is an indispensable coenzyme *in vivo* [16], and it plays an important role in tryptophan metabolism. It can upregulate L-kynurenine hydrolase (KYNU), which significantly downregulates inflammatory cytokine levels and reduces inflammation by affecting the KYN pathway [17, 18]. Studies have also found that tryptophan metabolism is associated with IDO1 [19]. IDO1 is a soluble protein secreted by adipose-derived mesenchymal stem cells, which inhibits local tissue inflammation and the autoimmune response [20]. Cell proliferation can be affected by the supply of nutrients, and the proliferation of T cells depends on the tryptophan supply. The expression of IDO1 can lead to depletion of tryptophan in the T cell microenvironment, leaving the cells in a state of tryptophan deficiency, which inhibits T cell proliferation.

In addition, the tryptophan catabolic pathway creates an immunosuppressive environment through the accumulation and secretion of tryptophan catabolic metabolites, such as kynurenine, 3-hydroxyanthranilic acid, and picolinic acid, key mediators of cellular immunosuppression of tryptophan [21]. These metabolites can directly inhibit T cell function, which leads to nonreactive T cells. Further, the effect of TLRs on A-MSCs is another approach to alleviate the immune response [22].

TLRs play an important role in the immunosuppressive function of A-MSCs. This function indicates that a variety of inflammatory and immune-mediated diseases can be treated [15]. They are involved in the initial recognition of microbial pathogens and pathogen-related components, especially TLR3 and TLR4 [23]. Studies have shown that TLR3- or TLR4-activated MSCs may regulate the Notch signalling pathway and upregulate Delta-like1 (DL1) to enhance the proliferation of Tregs [6]. Furthermore, it has been proven that activation of TLR6 in MSCs can increase the proliferation of peripheral blood leukocytes (PBLs) and enhance the release of lactate dehydrogenase MSCs, which confirmed the role of TLR6 in promoting the immunogenicity of MSCs [24]. Downregulation of TLR6 expression enhances lymphocytes inhibition and reduces the immune response. The occurrence of autoimmune diseases can decline under the lower immunogenicity of A-MSCs.

In our study, PLP (50 ng/mL) could upregulate TLR3 and TLR4 in A-MSCs, enhancing downstream NF-*κ*B expression. Activation of the TLR4/NF-*κ*B pathway results in increased secretion of downstream inflammatory factors, producing a proinflammatory effect, while high expression of the TLR3 receptor results in increased secretion of inflammatory inhibitory factors. However, the combined effects of these two receptors determine the immune regulation of A-MSCs. Therefore, TLR3 and TLR4 specific inhibitors were used to block TLR3 and TLR4 expression. The reduced CD3^+^CD8^+^ T lymphocyte inhibition after blocking TLR3 indicated an important role of TLR4. However, the inhibition of CD3^+^CD8^+^ T lymphocytes after blocking the TLR3 receptor alone was stronger, indicating that TLR3 is dominant. When TLR3 and TLR4 were simultaneously inhibited, the proportion of CD3^+^CD8^+^ T lymphocytes was significantly reduced compared with that of the control group. TLR3 and TLR4 are simultaneously activated under PLP stimulation; however, the role of TLR3 may be more advantageous than TLR4, leading to the final immunosuppressive effect at this concentration. The difference in TLR activation in A-MSCs by PLP may result in the decreased proportion of CD3^+^CD8^+^ T lymphocytes. In addition to enhancing expression of TLRs, PLP increases IDO1 secretion. PLP can increase the metabolism of tryptophan and promote the production of tryptophan metabolites [21]. In examinations of kynurenic acid, the concentration in supernatants of T lymphocytes cocultured with A-MSCs stimulated by various concentrations of PLP, and the concentration of kynurenic acid reached a maximum at 50 ng/mL and 100 ng/mL. There was no significant difference at 1000 ng/mL and 50 ng/mL, showed cytotoxicity at high concentrations and could not be used to upregulate immune regulation.

The interaction between IDO1 secretion and TLR expression may form a microenvironment, where CD3^+^CD8^+^ T lymphocytes do not adapt to survive and proliferation, thus reducing the inflammatory response of this microenvironment. Excessive concentrations of PLP may be toxic and have a negative impact on A-MSC survival. Therefore, the appropriate amount of PLP can enhance tryptophan metabolites and immunomodulatory function of A-MSCs.

## 5. Conclusion

In conclusion, this further clarified the mechanism and role of PLP in the immunoregulatory function of A-MSCs. Specifically, changes in TLR expression suppressed immunogenicity and the autoimmune response of A-MSCs. Further, increased IDO1 secretion promoted A-MSCs to inhibit lymphocytic proliferation, which are all due to the effect of PLP. Thus, PLP is a potential candidate to treat autoimmune diseases. Additional studies regarding proper application are required.

## Figures and Tables

**Table 1:** Differentiation culture medium.

Medium	Composition
Osteogenesis	Low-glucose DMEM (Gibco, USA)
	10% FBS (Gibco, USA)
	0.1 *μ*M dexamethasone (Solarbio, China)
	50 mM *β*-glycerol phosphate (Solarbio, China)
	0.2 mM ascorbic acid (Sigma-Aldrich, USA)

Adipogenesis	High-glucose DMEM (Gibco, USA)
	10% FBS (Gibco, USA)
	0.25 mM 3-isobutyl-1-methylxanthine (Solarbio, China)
	0.1 *μ*M dexamethasone (Solarbio, China)
	0.1 mM indomethacin (Sigma-Aldrich, USA) 6.25 *μ*g/mL insulin (PeproTech, UK)

Chondrogenesis	High-glucose DMEM (Gibco, USA)
	10% FBS (Gibco, USA)
	100 mg/mL sodium pyruvate (Solarbio, China)
	0.5 *μ*M dexamethasone (Solarbio, China)
	50 *μ*g/mL ascorbic acid (Sigma-Aldrich, USA)
	50 *μ*g/mL proline (Solarbio, China)
	10% insulin-transferrin-sodium selenite media supplement (ITS) (Sigma, USA)
	10 ng/mL transforming growth factor *β* (TGF-*β*) (GenScript, China)

**Table 2:** Primer sequence used in qPCR.

Gene	Primer sequence		Product length (bp)
GAPDH	F	5′-GCACCGTCAAGGCTGAGAAC	138
	R	5′-TGGTGAAGACGCCAGTGGA	

IDO1	F	5′-TGGCCAGCTTCGAGAAAGAG	157
	R	5′-TGGCAAGACCTTACGGACATC	

TLR3	F	5′-GCAAAAGATTCAAGGTACATCATGC	127
	R	5′-CCTCTTCGCAAACAGAGTGC	

TLR4	F	5′-TTATCACGGAGGTGGTTCCTA	102
	R	5′-TCAGGTCCAGGTTCTTGGTTG	

TLR6	F	5′-TAGGATAGCCACTGCAACATCA	113
	R	5′-CCGTCGGAGAACTGGATTCTG	

NF-*κ*B	F	5′-TTTTCGACTACGCGGTGACA	73
	R	5′-TCCTGCACAGCAGTGAGATG	

## Data Availability

The data used to support the findings of this study are available from the corresponding author upon request.

## Supplementary Materials

Supplementary 1 Supplementary Figure 1: morphology and pleiotropic differentiation ability of primary A-MSCs. A: Oil Red O staining. B: Alizarin red. C: cultured cells exhibiting a spindle-forming, fibroblast-like morphology. D: Alcian blue stain. E: Safranin O Green Cartilage Stain. F: control group cells remained with MSC morphology and negative staining marks.

Supplementary 2 Supplementary Figure 2: proportion of CD3^+^CD8^+^ T lymphocytes stimulated by 0 ng/mL, 2 ng/mL, 4 ng/mL, 6 ng/mL, 8 ng/mL, and 10 ng/mL PLP. The proportion of CD3^+^CD8^+^ T lymphocytes was significantly higher than those cocultured with A-MSCs. There was no difference between low concentrations.

Supplementary 3 Supplementary Figure 3: proliferation index of CD3^+^CD8^+^ T lymphocytes stimulated by 0 ng/mL, 2 ng/mL, 4 ng/mL, 6 ng/mL, 8 ng/mL, and 10 ng/mL PLP. The proliferation of CD3^+^CD8^+^ T lymphocytes was significantly higher than those cocultured with A-MSCs. There was no difference between low concentrations.

Supplementary 4 Supplementary Figure 4: apoptosis ratio of CD3^+^CD8^+^ T lymphocytes stimulated by of 0 ng/mL, 2 ng/mL, 4 ng/mL, 6 ng/mL, 8 ng/mL, and 10 ng/mL PLP. The proportion of CD3^+^CD8^+^ T lymphocytes was significantly higher than those cocultured with A-MSCs. There was no difference between low concentrations.

Supplementary 5 Supplementary Figure 5: live cell counts of A-MSCs stimulated by 0 ng/mL, 10 ng/mL, 20 ng/mL, 50 ng/mL, 100 ng/mL, and 1000 ng/mL PLP. The number of living A-MSCs increased significantly at 20 ng/mL, 50 ng/mL, and 100 ng/mL PLP compared to 0 ng/mL.
